# Comprehensive study of liposome-assisted synthesis of membrane proteins using a reconstituted cell-free translation system

**DOI:** 10.1038/srep18025

**Published:** 2015-12-15

**Authors:** Tatsuya Niwa, Yoshihiro Sasaki, Eri Uemura, Shugo Nakamura, Minato Akiyama, Mitsuru Ando, Shinichi Sawada, Sada-atu Mukai, Takuya Ueda, Hideki Taguchi, Kazunari Akiyoshi

**Affiliations:** 1 Department of Biomolecular Engineering, Graduate School of Biosciences and Biotechnology, Tokyo Institute of Technology, Midori-ku, Yokohama 226-8501, Japan; 2 Department of Polymer Chemistry, Graduate School of Engineering, Kyoto University, Katsura, Nishikyo-ku, Kyoto, 615-8510, Japan; 3 Department of Biotechnology, The University of Tokyo, 1-1-1 Yayoi, Bunkyo-ku, Tokyo, Japan; 4 Japan Science and Technology Agency (JST), The Exploratory Research for Advanced Technology (ERATO), Bio-nanotransporter Project, Katsura Int’tech Center, Katsura, Nishikyo-ku, Kyoto 615-8530, Japan; 5 Department of Medical Genome Sciences, Graduate School of Frontier Sciences, University of Tokyo, FSB401, 5-1-5 Kashiwanoha, Kashiwa, Chiba 277-8562, Japan

## Abstract

Membrane proteins play pivotal roles in cellular processes and are key targets for drug discovery. However, the reliable synthesis and folding of membrane proteins are significant problems that need to be addressed owing to their extremely high hydrophobic properties, which promote irreversible aggregation in hydrophilic conditions. Previous reports have suggested that protein aggregation could be prevented by including exogenous liposomes in cell-free translation processes. Systematic studies that identify which membrane proteins can be rescued from irreversible aggregation during translation by liposomes would be valuable in terms of understanding the effects of liposomes and developing applications for membrane protein engineering in the context of pharmaceutical science and nanodevice development. Therefore, we performed a comprehensive study to evaluate the effects of liposomes on 85 aggregation-prone membrane proteins from *Escherichia coli* by using a reconstituted, chemically defined cell-free translation system. Statistical analyses revealed that the presence of liposomes increased the solubility of >90% of the studied membrane proteins, and ultimately improved the yields of the synthesized proteins. Bioinformatics analyses revealed significant correlations between the liposome effect and the physicochemical properties of the membrane proteins.

Structural and functional characterization of membrane proteins involved in ion transport, signal transduction, energy production, and cellular communication, for example, is an important topic in protein engineering, pharmaceutical science, and for constructing nanodevices such as nanocarriers in advanced drug delivery systems^1^,^2^,^3^,^4^,^5^,^6^. One of the main barriers to such research is the effective production of a sufficient amount of homogeneous membrane proteins in cell-based systems owing to the low yield, poor solubility, and difficulties in purifying proteins, and the overexpressed exogenous proteins may be toxic to the host cells^7^,^8^. Cell-free protein synthesis is a promising alternative method that can overcome the limitations of conventional cell-based methods, because it offers a simple, flexible, and chemically defined approach for the rapid production of proteins^9^,^10^,^11^,^12^,^13^. However, moving away from cell-based systems has introduced another problem, the difficulty of handling membrane proteins in an aqueous environment because membrane proteins do not dissolve or disperse in water. For this reason, many conventional biophysical and biochemical protocols cannot be used, and this complicates the purification and handling of membrane proteins. In cells, membrane proteins usually exist in lipid bilayer membranes. Therefore, appropriate interactions between lipids and the proteins are required to facilitate correct and functional folding of membrane proteins during synthesis^14^,^15^.

Amphiphilic materials such as detergents micelles^16^, amphipols^17^, bicelles^18^, nanodiscs^19^, and microsomes^20^ can mimic the membrane environment and have been used to facilitate the production of membrane proteins in soluble forms. Translation of membrane proteins in the presence of liposomes as an artificial cell membrane seems to be straightforward and attractive approach for cell-free systems. Several reports have described successful cell-free expression of several membrane proteins in the presence of liposomes. These proteins included stearoyl-CoA desaturase^21^, bacteriorhodopsin^22^ (a voltage-dependent anion channel), and the proapoptic protein Bak^23^, which were expressed using *Escherichia coli* or wheat germ extract cell-free protein synthesis systems (reviewed in^24^). We have also reported that some expressed membrane proteins such as apo-cytochrome b5^25^, connexin 43^26^,^27^ or bacteriorhodopsin^28^ were directly incorporated into liposomes. Using a liposome-chaperoned cell-free synthesis (LCC) system, the liposomes prevented the irreversible aggregation of hydrophobic membrane proteins, and aided their correct folding and oligomerization within the liposomal lipid bilayer membranes. In addition, we have demonstrated that connexin 43-integrated proteoliposomes had the potential to transfer small molecules to the cytoplasm directly, and thus represented a novel drug delivery system^26^.

The LCC system has some advantages over approaches using other membrane mimicking supplements. For example, high throughput screening of pharmaceutically or biologically important membrane proteins against ligand libraries is possible owing to the technical simplicity of protocols based on this system. In this study, we sought to elucidate the general versatility of the LCC system and to examine the effects of liposomes on membrane protein integration. To achieve this, we examined the expression of 85 membrane proteins from *E. coli* in a reconstituted cell-free translation system, which only contained the factors essential for protein synthesis. This was necessary to evaluate the effects of liposomes in uniform conditions, because debris, which is often present in other translation systems, obstructs the direct analysis of the effects of liposomes. For this purpose, we used an *E. coli* reconstituted cell-free system, the protein synthesis using recombinant element (PURE) system^29^,^30^,^31^. Using the PURE system, we previously analyzed the aggregation properties of all water-soluble *E coli* proteins in chaperone-free conditions^31^. The PURE system was also used to investigate the effect of chaperones such as GroEL and DnaK^32^,^33^,^34^,^35^ and an artificial chaperone^36^ on newly synthesized cytoplasmic proteins. More recently, we have synthesized several membrane machineries involved in vital cellular functions using the PURE system, and analyzed their functions within liposomes^37^,^38^,^39^.

In this study, we extended the results of our prior studies by comprehensively evaluating the effects of liposomes on the translation of 85 membrane proteins from *E. coli* by using the PURE system. Figure 1a summarizes the systematic approach, which involved the synthesis of individual membrane proteins in the presence of liposome, quantification of the solubility of these proteins in a centrifugation-based assay, and statistical analyses of the obtained data. This “cell-free proteome” approach^35^, in which the translation properties of relatively large number of membrane proteins are examined individually in the same cell-free translation conditions, are vital to validate the general applicability of the LCC system. This technique also enables researchers to evaluate specific membrane proteins, which are ordinarily expressed at very low levels in cells. The statistical analyses performed in this study revealed some intriguing results in terms of the production of membrane proteins and the relationship between the enhanced solubility in the presence of liposomes and the physicochemical properties of membrane proteins.

## Results

### Aggregation analysis in liposome-chaperoned cell free system

**Figure 1:**
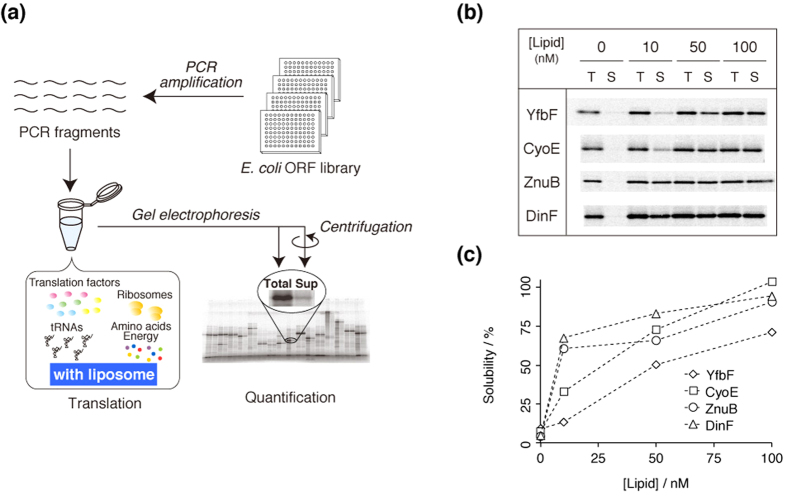
Schematic illustration of the experiment and representative results. (**a**) Schematic illustration of the cell-free protein synthesis system. Membrane proteins were individually expressed with a reconstituted cell-free translation system, the PURE system, in the absence or presence of liposomes. Each translation product was labeled with [^35^S]-methionine. After translation, the uncentrifuged total fraction (Total) and the supernatant fraction after centrifugation (Sup) were subjected to SDS-PAGE and quantified by autoradiography. The ratio of the translation products in the Total and Sup fractions was defined as the solubility, which represents the protein’s aggregation propensity. The data obtained in this experiment were analyzed statistically. (**b**) SDS-PAGE of four *E. coli* membrane proteins (YfbF, CyoE, ZnuB, and DinF) synthesized in the absence or presence of liposomes. (**c**) Liposome concentration-dependence of the solubility of four translated membrane proteins.

Initially, four membrane proteins (YfbF, CyoE, ZnuB, and DinF), which were randomly selected from the *E. coli* integral membrane protein library, were synthesized in the cell-free PURE system at 37 °C for 120 min in the absence or presence of liposomes. Some of the properties of these proteins are summarized in Table S1. Each protein was synthesized in the absence and presence of liposomes and the translated protein solution was analyzed by sodium dodecyl sulfate–polyacrylamide gel electrophoresis (SDS-PAGE). The expression levels of translated proteins labeled with [^35^S]-methionine was quantified by autoradiography. The aggregation propensity was examined using a centrifugation assay. Briefly, the soluble fractions were separated from an aliquot of the translation mixture by centrifugation, and each fraction was subjected to SDS-PAGE to quantify the total and soluble protein fractions (Fig. 1b,c). The net productivity of the PURE system was calculated as the autoradiographic intensity of the total fraction. Protein solubility was defined as the proportion of the expressed protein in the supernatant fraction to that in the uncentrifuged total fraction. As shown in Fig. 1b, the protein abundance in the soluble fraction in the absence of liposome was very low, presumably due to the hydrophobic properties of the membrane proteins. However, the protein abundance in the soluble fraction was significantly increased for all four proteins when the cell-free synthesis was performed in the presence of liposomes (Fig. 1b,c). The solubility of the proteins increased proportionally to the concentration of liposomes in the PURE system (Fig. 1b,c). These results suggest that liposomes prevented the aggregation of membrane proteins and solubilized the proteins by incorporating the synthesized proteins into the hydrophobic lipid bilayer membranes.

### Effects of liposomes on protein synthesis

Next, we analyzed the solubilization effects of liposomes on a larger set of 85 randomly selected *E. coli* integral membrane proteins. All the selected membrane proteins were annotated as typical α-helical integral membrane proteins, not β-barrel membrane proteins. This set included anionic, cationic, and neutral membrane proteins, and the molecular weight ranged from 8.2 to 146.5 kDa (Figure S1). There was no obvious bias in protein selection in terms of the basic physicochemical properties of the membrane proteins, including the molecular weight, number of transmembrane domains, and isoelectric point (p*I*).

**Figure 2:**
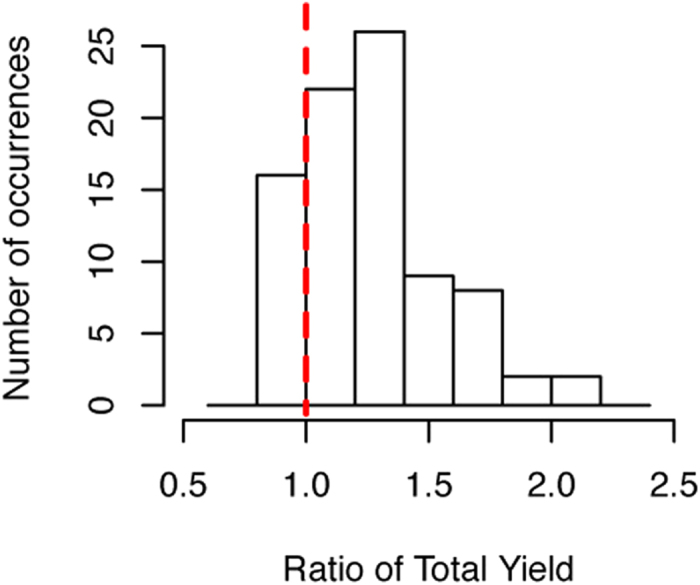
Histogram of the ratio of the total synthetic yield of 85 translated membrane proteins. The ratio of the synthetic yield of proteins was calculated by dividing the synthetic yield in the presence of 100 nM of liposomes by that in the absence of liposomes. The blue dashed line indicates a ratio of 1.0.

The solubility and the synthetic yield of each protein were quantified in the absence or presence of 100 nM liposome. All the evaluated data were shown in Table S2. The experimental error (defined as the standard deviation, *SD*) of the assay was previously estimated to be 10%^31^,^34^. In the present study, the *SD* of the solubilities both in the presence and absence was <10%, which means the procedure was reproducible as in previous studies^31^,^34^. Our first question was whether liposomes facilitate protein synthesis in a cell-free system. As shown in Fig. 2 and S2, the total amount of translated proteins, which represents the productivity of the PURE system, was improved by the addition of liposomes. The total expression of 80% (68/85) of the translated membrane proteins was increased when they were expressed in the presence of liposomes, and the largest increase in expression was more than two-fold. This result is clear contrast with that obtained for translation of cytoplasmic proteins^35^,^36^. In our previous study, the presence of chaperones, such as DnaK and GroEL/ES, hardly affected the yield of translated proteins in the PURE system^35^. Likewise, polysaccharide nanogels, which display an artificial chaperone function by incorporating aggregation-prone proteins within their hydrogel matrix to inhibit irreversible aggregation, did not markedly affect the production of translated proteins in the PURE system^36^.

### Systematic analysis of protein aggregation in the liposome-chaperoned cell-free system

**Figure 3:**
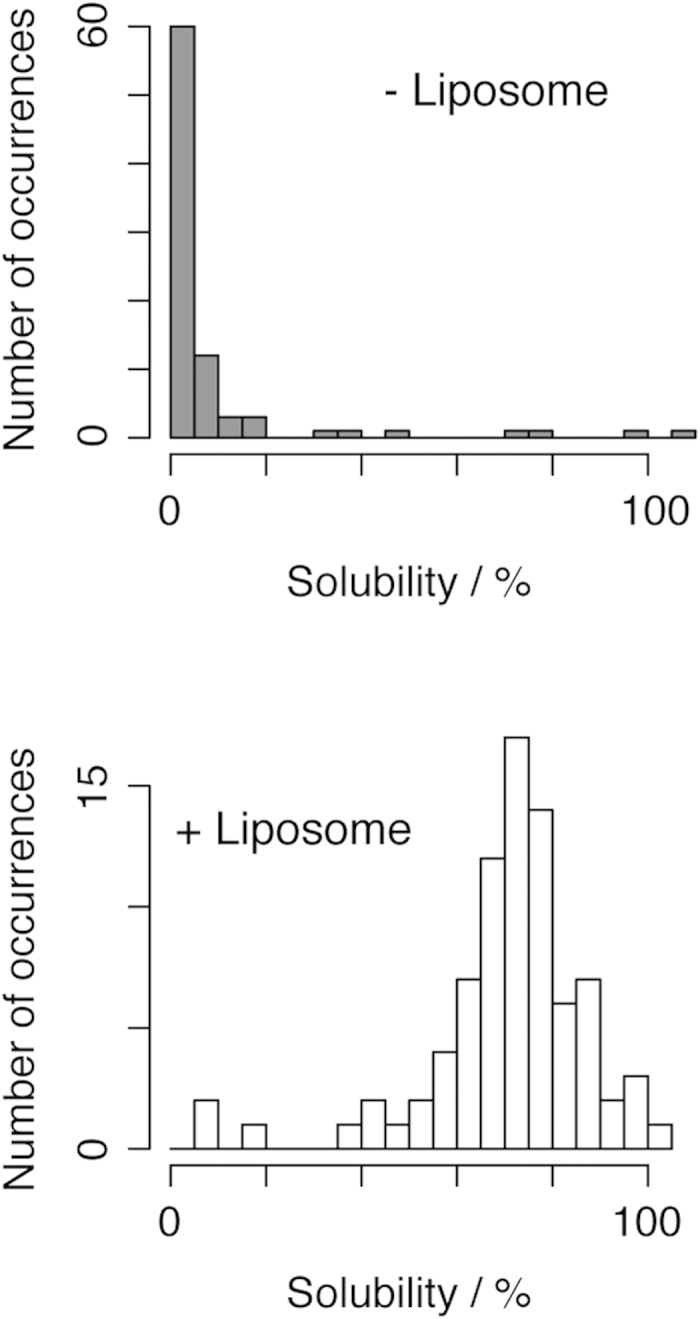
Histograms of the solubility of the translated membrane proteins in the absence and presence of liposomes. The upper panel shows the solubility distribution in the absence of liposomes and the lower panel shows the solubility distribution in the presence of 100 nM of liposomes. Solubility was defined as the amount of protein in the supernatant fraction divided by the amount of protein in the uncentrifuged total fraction.

The membrane proteins examined in this study formed aggregates when they were expressed without liposomes (Fig. 3). Although four membrane proteins were soluble (>70% solubility) even in the absence of liposomes, the solubility of 71% (60/85) of the translated membrane proteins was <5% owing to their formation of insoluble aggregates. The analyses showed that liposomes increased the solubility of the aggregation-prone membrane proteins (Fig. 3), indicating that liposomes associate with most membrane proteins during or after translation to prevent the irreversible aggregation of these proteins (Fig. 3). Of note, the solubility of 92% (78/85) of the tested membrane proteins was >50% in the presence of liposomes, which indicates that liposomes have strong solubilization effects on membrane proteins translated using the PURE system.

### Relationship between solubility and physicochemical properties

**Figure 4:**
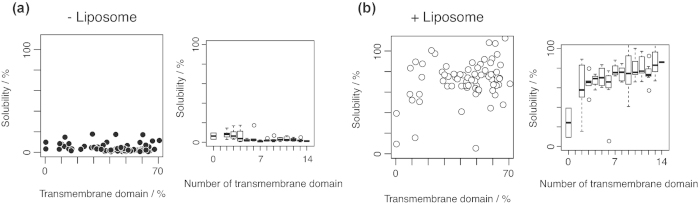
Correlation between protein solubility and properties of the transmembrane domains for 78 proteins in the absence (**a**) or presence (**b**) of liposomes. The scatterplots in the left panels shows the correlation between protein solubility and the length of the transmembrane domain relative to the total amino acid length. The boxplots in the right panel show the solubility of the proteins according to the number of transmembrane helices.

We next determined whether the increased solubility of the membrane proteins in the presence of liposomes was correlated with the physicochemical properties of the proteins. In our previous study in which we examined potential chaperone-mediated effects on the aggregation of cytoplasmic proteins, we defined aggregation-prone proteins as those with a solubility of <30%^35^. Based on this definition, seven membrane proteins in the present study had solubilities of >30% in the absence of liposome, and were excluded from the analyses. To examine likely processes by which liposomes prevent membrane protein aggregation, we determined the correlations between solubility and the physicochemical properties of proteins, including the molecular weight, the deduced p*I*, and the total protein yield. However, the overall correlations between these variables and solubility in the presence of liposome were quite poor (Figure S3). However, we found a moderate bias when the data were plotted against the percentage of transmembrane domains in the peptide sequence of each membrane protein (Fig. 4). Figure 4b shows that some proteins were biased in the upper right area, whereas the solubilities shown in the left area were weakly correlated with the percentage of transmembrane domains. These findings suggest that liposomes have greater solubilization effects on membrane proteins with higher ratios of transmembrane domains. Similar tendencies were observed when we compared the median solubilities for each number of transmembrane domains in the presence or absence of liposomes (Fig. 4a,b, right panel).

**Figure 5:**
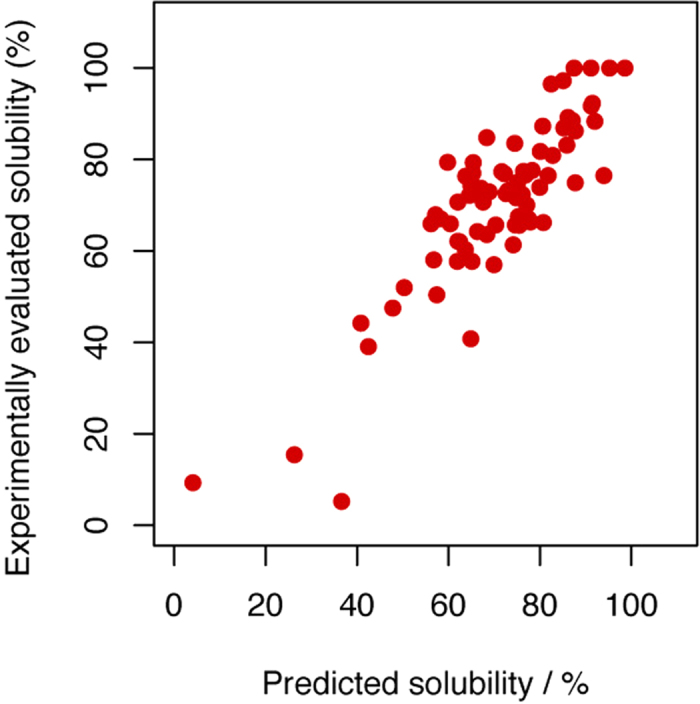
Comparison between the solubility predicted using the multiple regression model and the experimentally determined solubility in the presence of liposomes. The multiple regression model was developed by stepwise selection of 18 variables (see main text and Methods). The variables included in the model are listed in Table 1.

We then conducted a further analysis of other structural properties, including the amino acid frequency, AAindex^40^, disorder tendency, secondary structural parameters, number of residue–residue interactions, and the contact order (a property related to protein structure and folding). The structural parameters were predicted from the amino acid sequences, as described in the data analysis section. We first compared the correlation coefficients between each variable and the solubility of proteins in the presence of liposomes, and 77 variables with high correlation coefficients were selected for further analysis. Then, to identify the variables with strong associations with protein solubility in the presence of liposome, we used a stepwise multiple linear regression method. This method determines the set of variables that is suitable for a good regression model by adding and deleting a variable one by one to minimize AIC (Akaike’s Information Criterion^41^). Using this approach, 18 variables were found to be independently associated with the solubilization effects of liposomes (Table 1). These variables described the properties related to regions of the protein inside or outside the membrane, and suggest that the degree of solubilization in the presence of liposomes was influenced by amino acid sequences or structures of the region outside the membrane in particular. In addition, the predicted solubility calculated by the multiple linear regression model constructed using these 18 variables showed a strong correlation with the solubility determined experimentally (*r* = 0.867, Fig. 5 and Table S3). Furthermore, to estimate the significance of these features to an independent dataset, we divided all samples into six parts randomly, and the regression analysis was applied to each combination of five parts (i.e. one part is eliminated). We repeated this procedure 50 times, 300 trials were conducted in total, and the frequency of extraction of each feature was counted. Among 18 features selected by the stepwise multiple linear regression analysis, 14 features were included in the members of top 18 features (Table S4). These results suggest that the features extracted by the stepwise multiple linear regression analysis also have a strong contribution to the effect of liposome for an independent dataset.

## Discussion

In this study, we systematically analyzed the effects of liposomes on the solubility of 85 aggregation-prone membrane proteins from *E. coli* in a reconstituted cell-free translation system, the PURE system. The present results clearly showed that liposomes enhanced the translation of many membrane proteins, and prevented irreversible aggregation of most of these proteins. Of note, expression in the presence of liposomes increased the production yields of 80% of the translated membrane proteins. A plausible explanation for the increased yield in the presence of liposomes is that membrane proteins often show strong interactions with the exit tunnel of the ribosome, which might cause a severe delay in translation. Liposomes might abolish this interaction because of their extremely high affinity for transmembrane domains. Another possibility is that liposomes might prevent interactions between partially translated hydrophobic polypeptides or prevent co-aggregation, both of which halt translation.

Notably, we also found some biases in the solubilities of proteins expressed in the liposome-chaperoned cell-free translation system. In particular, the increased solubility in the presence of liposomes was associated with the number of transmembrane domains. The correlation between the number of transmembrane domains and the solubility of the protein in the presence of liposomes is reasonable because the transmembrane domains not only play an important role in protein aggregation because of the hydrophobicity of these domains, but they are also thought to show high affinity for lipid bilayer membranes. A previous study, which investigated the solubilization effects of liposomes in a wheat germ cell-free system, suggested that the solubility of proteins with one transmembrane domain was lower than that of proteins with more than two transmembrane domains^42^. The current results support the hypothesis that the increased solubility in the LCC system is dependent on the number of transmembrane domains. In addition, the statistical analysis revealed that the properties of regions of the protein outside the membrane influenced the solubilization effects of liposomes, and suggests that the properties of the non-transmembrane region may affect the affinity for liposomes.

Although we have proposed several mechanisms to describe the effects of liposomes on protein solubility, it is difficult to provide a clear explanation for the dependence of liposomes in the LCC system based on the available data. One of the plausible explanations is that liposome could rescue apparently hydrophobic membrane proteins with a relatively large number of transmembrane domains via hydrophobic interactions with the hydrophobic lipid membrane. By contrast, other factors, including electrostatic interactions, are necessary to rescue less hydrophobic membrane proteins with few transmembrane domains. To confirm this possibility, we are now performing studies to examine the effects of properties of liposomal membranes, including phase transition, surface charge, and size on the solubilization of expressed proteins. In addition, because we selected only typical α-helical integral membrane proteins in this study, we cannot mention whether the biases and correlations observed in the α-helical membrane proteins were also observed in the β-barrel membrane proteins.

In conclusion, this study is unique in providing an explicit *in vitro* experimental demonstration of the role of liposomes in preventing the aggregation of membrane proteins during translation. At this time, we cannot exclude the possibility that the soluble fraction might contain some oligomeric proteins, which were not precipitated by the ultracentrifugation conditions. It is also unclear whether all membrane proteins employed here were in correctly folded native forms when they interacted with the liposome, though we have already confirmed that some membrane proteins synthesized using the cell-free protein synthesis system in the presence of liposomes correctly folded to its native forms and shows their functions within the lipid bilayer membrane^26^,^27^,^28^. Despite these limitations, the results of this study provide invaluable support for membrane protein research.

## Methods

### Materials

The genes encoding 85 proteins were individually amplified by polymerase chain reaction (PCR) using universal primers and plasmids from an *Escherichia coli* open reading frame (ORF) library (ASKA library)^31^,^35^,^43^. These PCR products were used as the templates in the cell-free protein synthesis reaction without further purification. All of the chemicals were of analytical grade and were used without further purification. Nuclease-free water (Millipore Co., Billerica, MA, USA) was used throughout this study. The transcription–translation-coupled reactions were performed using PURE*frex* 1.0 (GeneFrontier Corporation, Japan), according to the manufacturer’s instructions.

### Liposome preparation

Liposomes were prepared using an established method^28^. Briefly, an appropriate amount of 1,2-dioleoyl-sn-glycero-3-phosphocholine (DOPC) (Nichiyu, Tokyo, Japan) was dissolved in chloroform. The solvent was evaporated under argon gas flow and the residual trace solvent was completely removed *in vacuo* to yield a thin film on the wall of a glass vial. The lipid film was hydrated by adding 50 mM HEPES buffer (pH 7.5) and vortexing the tube at a temperature above the phase transition temperature. The lipid suspension was extruded through a polycarbonate filter with 100-nm pores. The lipid concentrations of the liposomes were adjusted to 50 mM.

### Cell-free protein synthesis and centrifugation-based aggregation assay

The procedure for evaluating the protein aggregation propensity was based on a previously reported method^31^,^35^ with slight modifications. Each ORF was individually translated with the PURE system in the absence or presence of liposomes. To detect the expressed proteins, [^35^S]-methionine was added to the PURE system^31^,^35^. Protein synthesis was performed at 37 °C for 120 min. To remove the background signal derived from [^35^S]-methionyl-tRNA, 0.04 mg/ml of RNase A was added to the solution after the reaction. Then, an aliquot was withdrawn as the total fraction. The remainder was centrifuged at 20,000 × *g* for 30 min, and the supernatant was collected. The total and supernatant fractions were separated by SDS-PAGE, and the band intensities were quantified by autoradiography. The samples were not boiling at 95 °C for 5 min before SDS-PAGE because many membrane proteins formed large insoluble aggregates in these conditions, and they could not be separated by SDS-PAGE after boiling. The solubility of the protein, an index of protein aggregation propensity, was calculated as the ratio of the supernatant to the total protein^35^.

### Data analysis

The molecular weight, p*I*, and amino acid content were calculated from the amino acid sequences obtained from GenoBase (http://ecoli.naist.jp/GB8/). Τransmembrane domains were predicted using TMHMM ver. 2.0 (http://www.cbs.dtu.dk/services/TMHMM/). Stepwise multiple linear regression analysis was conducted as follows. As explanatory variables, 943 variables, including the frequencies of the 20 amino acids, the amino acid index (AAindex^40^), frequencies of rare codons, disorder tendency predicted by DISOPRED ver 3^44^, and structural parameters such as the predicted secondary structure and contact order predicted by CRNPRED^45^, were determined for each protein. To exclude potential multicollinearity, explanatory variables with high correlation coefficients >0.7 with other variables were omitted. After applying this method, 77 variables showing high correlation coefficients with protein solubilities in the presence of liposome were selected for further analyses. These 77 variables were subjected to stepwise multiple linear regression analysis to select subsets of variables and obtain a model with the lowest Akaike’s information criterion^41^. The stepwise multiple linear regression was conducted by using “step” function in R software^46^ (http://www.R-project.org).

## Additional Information

**How to cite this article**: Niwa, T. *et al*. Comprehensive study of liposome-assisted synthesis of membrane proteins using a reconstituted cell-free translation system. *Sci. Rep*. **5**, 18025; doi: 10.1038/srep18025 (2015).

## Supplementary Material

Supplementary Information

Supplementary Table S2

Supplementary Table S3

## Figures and Tables

**Table 1:** Variables showing strong correlations with the solubilities of proteins in stepwise multiple linear regression analysis.

Variable ID	Location	Description	*t*-value	*P*-value
in_fP	Inside	Relative number of Pro residues	−5.199	2.63×10^-6^***
n_out	Outside	Number of amino acid residues	4.678	1.73×10^-5^***
fS	All	Relative number of Ser residues	4.177	9.88×10^-5^***
in_fM	Inside	Relative number of Met residues	3.578	0.000699***
ave_len_out	Outside	Average loop length	−3.32	0.001549**
in_fD	Inside	Relative number of Asp residues	3.074	0.003196**
in_fL	Inside	Relative number of Leu residues	2.85	0.006014**
in_fS	Inside	Relative number of Ser residues	−2.58	0.012382*
tmh_fC	TM helix	Relative number of Cys residues	2.383	0.020428*
fR	All	Relative number of Arg residues	−2.313	0.024227*
in_fF	Inside	Relative number of Phe residues	2.257	0.027698*
tmh_fH	TM helix	Relative number of His residues	−2.245	0.02853*
in_dsALL2	Inside	Disorder tendency	−2.125	0.037798*
in_fT	Inside	Relative number of Thr residues	2.125	0.03781*
out_fN	Outside	Relative number of Asn residues	−2.008	0.04921*
out_fG	Outside	Relative number of Gly residues	−1.939	0.057321
AURR980101_rel	All	Normalized positional residue frequency at helix termini N4′^47^	1.717	0.091165
out_fR	Outside	Relative number of Arg residues	1.715	0.091682

The positions of amino acids (inside/outside/transmembrane helix) were predicted using TMHMM, as described in the data analysis section.

TM, transmembrane.

*P < 0.01; **P < 0.01, and ***P < 0.001.
