# Supplementary material for: Comprehensive study of liposome-assisted synthesis of membrane proteins using a reconstituted cell-free translation system
Source: Sci Rep. 2015 Dec 15;5:18025. doi: 10.1038/srep18025 (PMC4678891; doi:10.1038/srep18025)
Supplement: Supplementary Information [file srep18025-s1.doc]

Supplementary Information

**Comprehensive study of liposome-assisted synthesis of membrane proteins using a reconstituted cell-free translation system**

Tatsuya Niwa^1^, Yoshihiro Sasaki^2^, Eri Uemura^1^, Shugo Nakamura^3^, Minato Akiyama^2^, Mitsuru Ando^2,4^, Shinichi Sawada^2,4^, Sada-atu Mukai^2,4^, Takuya Ueda^5^, Hideki Taguchi^1^ and Kazunari Akiyoshi^2,4^

^1^Department of Biomolecular Engineering, Graduate School of Biosciences and Biotechnology, Tokyo Institute of Technology, Midori-ku, Yokohama 226-8501, Japan

^2^Department of Polymer Chemistry, Graduate School of Engineering, Kyoto University, Katsura, Nishikyo-ku, Kyoto, 615-8510, Japan

^3^Department of Biotechnology, The University of Tokyo, 1-1-1 Yayoi, Bunkyo-ku, Tokyo, Japan

^4^Japan Science and Technology Agency (JST), The Exploratory Research for Advanced Technology (ERATO), Bio-nanotransporter Project, Katsura Int'tech Center, Katsura, Nishikyo-ku, Kyoto 615-8530, Japan

^5^Department of Medical Genome Sciences, Graduate School of Frontier Sciences, University of Tokyo, FSB401, 5-1-5 Kashiwanoha, Kashiwa, Chiba 277-8562, Japan

**Supplementary Table S1**

**Table S1.** Physicochemical properties of four membrane proteins

| Protein | *M*w /kDa | *N_TMD_* | p*I* | Function |
| --- | --- | --- | --- | --- |
| YfbF | 36.3 | 2 | 7.7 | undecaprenyl phosphate-l- Ara4FN transferase |
| CyoE | 32.2 | 7 | 10 | protoheme IX farnesyltransferase |
| ZnuB | 27.7 | 7 | 9.1 | zinc transporter subunit, membrane component of ABC superfamily |
| PgsA | 20.7 | 4 | 9.7 | phosphatidylglycerophosphate synthetase |

*Mw*, molecular weight; *N_TMD_*, number of transmembrane domains; p*I*, isoelectric point.

**Table S2** and **S3** are provided as Excel spreadsheets.

**Table S4. Variables showing strong correlations with the solubilities of proteins in stepwise multiple linear regression analysis**

| Variable ID | Location | Description | Rank | Frequency |
| --- | --- | --- | --- | --- |
| in_fP | Inside | Relative number of Pro residues | **3** | **258** |
| n_out | Outside | Number of amino acid residues | **2** | **263** |
| fS | All | Relative number of Ser residues | **10** | **180** |
| in_fM | Inside | Relative number of Met residues | **1** | **275** |
| ave_len_out | Outside | Average loop length | **5** | **234** |
| in_fD | Inside | Relative number of Asp residues | **12** | **178** |
| in_fL | Inside | Relative number of Leu residues | 40 | 133 |
| in_fS | Inside | Relative number of Ser residues | **7** | **211** |
| tmh_fC | TM helix | Relative number of Cys residues | **16** | **167** |
| fR | All | Relative number of Arg residues | 49 | 128 |
| in_fF | Inside | Relative number of Phe residues | **4** | **238** |
| tmh_fH | TM helix | Relative number of His residues | 20 | 160 |
| in_dsALL2 | Inside | Disorder tendency | **6** | **215** |
| in_fT | Inside | Relative number of Thr residues | **13** | **175** |
| out_fN | Outside | Relative number of Asn residues | **9** | **182** |
| out_fG | Outside | Relative number of Gly residues | **8** | **203** |
| AURR980101_rel | All | Normalized positional residue frequency at helix termini N4' (41) | 34 | 134 |
| out_fR | Outside | Relative number of Arg residues | **18** | **165** |

The positions of amino acids (inside/outside/transmembrane helix) were predicted using TMHMM, as described in the data analysis section.

TM, transmembrane.

Rank indicates the rank order of the frequency of extraction on the repetitive regression analysis.

Frequency indicates the frequency of extraction on the repetitive regression analysis.

Bold number indicates their ranks are above the 18th.

**Supplementary Figures S1-S3**

**Figure S1 |** Histograms of the basic properties (molecular weight, isoelectric point, number of transmembrane domains, and ratio of the number of transmembrane domains to protein length) for the 85 membrane proteins from *E. coli* that were examined in this study.

**Figure S2 |** Total yields of the translated membrane proteins obtained using the PURE system in the absence and presence of liposomes.

**Figure S3 |** Correlation between the solubility and basic properties of the 85 membrane proteins synthesized in the absence (upper panels) and presence (lower panels) of liposomes.
